# Expression of BMP-7 in human gastric cancer and its clinical significance

**DOI:** 10.1038/sj.bjc.6606075

**Published:** 2011-01-11

**Authors:** M Aoki, S Ishigami, Y Uenosono, T Arigami, Y Uchikado, Y Kita, H Kurahara, M Matsumoto, S Ueno, S Natsugoe

**Affiliations:** 1 Digestive Surgery, Surgical Oncology, Kagoshima University School of Medicine, 8-35-1 Sakuragaoka, Kagoshima 890-8520, Japan

**Keywords:** gastric cancer, BMP-7, nodal involvement, histology, prognosis

## Abstract

**Background:**

Bone morphogenetic protein-7 (BMP-7) is a signalling molecule belonging to the transforming growth factor--superfamily. Recent studies have demonstrated the clinical impact of BMP-7 expression in various human cancers. However, there have been few reports detailing this in gastric cancer.

**Methods:**

We immunohistochemically investigated the expression of BMP-7 in 233 gastric cancer patients to disclose the clinicopathological features of BMP-7-positive gastric cancer.

**Results:**

Immunohistochemically, in human gastric cancer, BMP-7 expression was identified in cellular membranes but also in the cytoplasm of cancer cells. Bone morphogenetic protein-7-positive expression was found in 129 of 233 patients (55%). Bone morphogenetic protein-7 expression was correlated with tumour size, nodal involvement, lymphatic invasion, venous invasion and histology (*P*<0.05). Bone morphogenetic protein-7 expression was significantly correlated with patient postoperative outcome, especially in the undifferentiated group. Multivariate analysis revealed BMP-7 expression as one of the independent prognostic factors next to the depth of invasion and nodal involvement (*P*<0.01).

**Conclusions:**

From the data collected, it would be appropriate to conclude on the possible regulation of gastric cancer progression by autocrine or paracrine BMP-7 loops. We can use BMP-7 expression as one of the strong predictors of risk of tumour recurrence in gastric cancer.

Gastric cancer is the fourth most common cancer. East Asian countries in particular, including Japan, are the most high-risk areas for gastric cancer. In Japan, the establishment of mass screening by photo fluoroscopy or gastrointestinal fibroscopy has led to gastric cancer being diagnosed asymptomatically and curatively treated with gastrectomy; the survival rate of gastric cancer has drastically improved. However, even for patients who have received curative surgery, the recurrence of gastric cancer occurs (Parkin *et al*, 2005; Brenner *et al*, 2009). In this context, it is necessary to find novel cancer-related factors to use as markers for diagnosis and treatment of gastric cancer.

Bone morphogenetic proteins (BMPs) are signalling molecules belonging to the transforming growth factor (TGF)--superfamily, with >30 subtypes in mammals, *Drosophila*, *Xenopus* and sea urchin (Wozney *et al*, 1988; Ducy and Karsenty, 2000). Bone morphogenetic proteins were originally identified as cytokines inducing ectopic chondro-osteogenesis and have a role in skeletal and joint morphogenesis, bone remodelling and fracture repair (Urist, 1965; Chen *et al*, 2004). However, the function of BMPs is linked not only to bone tissue, but also to cellular homeostasis and embryonic development. Several studies demonstrated that mice deficient in different BMPs exhibited impairments and abnormalities of various organs (Dudley *et al*, 1995; Luo *et al*, 1995; McPherron *et al*, 1997; Solloway and Robertson, 1999).

Expression of BMP-7 is highest in the kidney and it is thought to be related to kidney and eye development and skeletal patterning (Dudley *et al*, 1995; Luo *et al*, 1995). Moreover, BMP-7 reverses chronic renal injury by counteracting TGF-1-induced epithelial-to-mesenchymal transition (Zeisberg *et al*, 2003). Recent studies demonstrated that BMP-7 expression is found in various human cancers, and regulates cell differentiation, proliferation, migration, invasion and apoptosis (Andrews *et al*, 1994; Ro *et al*, 2004; Yang *et al*, 2005, 2006; Grijelmo *et al*, 2007; Alarmo *et al*, 2009). However, to our knowledge, there have been no reports demonstrating the association between BMP-7 expression and clinicopathological factors including prognosis in gastric cancer. Thus, the purpose of this study was to investigate BMP-7 expression by immunohistochemistry in gastric cancer and evaluate the clinical impact of BMP-7-positive gastric cancer.

## Materials and methods

### Detection of mRNA expression of *BMP-7* in gastric cancer cell lines

We used five gastric cancer cell lines (KATOIII, NUGC-4, MKN45, MKN74 and MKN7 (Riken Cell Bank, Tsukuba, Japan)) to detect mRNA expression of *BMP-7* by RT–PCR. First, five gastric cancer cell lines were cultured in RPMI-1640 (Sigma-Aldrich Co., St Louis, MO, USA) supplemented with antibiotics (100 units ml^–1^ penicillin and 100 *μ*g ml^–1^ streptomycin) and 10% fetal bovine serum (MBL, Nagoya, Japan) at 37 °C in a humidified atmosphere of 5% CO_2_ in air. Proliferated cells were used to detect mRNA expression of *BMP-7*. Total RNA was extracted from the cell lines using RN easy Mini-Kit (Qiagen, Valencia, CA, USA) according to the manufacturer's instructions. Complementary DNA (0.05 *μ*g *μ*l^–1^) was synthesised as described previously (Yokomakura *et al*, 2007). Amplification of *BMP-7* and *glyceraldehyde-3-phosphate dehydrogenase* (*GAPDH*) cDNA was performed in a total volume of 30 *μ*l, which included 1 *μ*l of the cDNA product sample, 0.5 *μ*M each of *BMP-7* primer (Roche, Mannheim, Germany) and *GAPDH* primer (forward primer: 5′-TTGGTATCGTGGAAGGACTCA-3′ and reverse primer: 5′-TGTCATCATATTTGGCAGGTTT-3′), GeneAmp 10 × PCR Buffer (Applied Biosystems, Foster City, CA, USA), 0.5 U AmpliTaq DNA polymerase (Applied Biosystems) and 25 mM dNTP mixture (Takara Bio Inc., Otsu, Japan). The *GAPDH* gene served as an internal control. The *BMP-7* and *GAPDH* amplification reactions were 30 cycles (*BMP-7*) or 26 cycles (*GAPDH*) of denaturation at 94 °C for 60 s (*BMP-7*) or 30 s (*GAPDH*), annealing at 64 °C for 60 s (*BMP-7*) or 54 °C for 30 s (*GAPDH*), and elongation at 72 °C for 60 s (*BMP-7*) or 30 s (*GAPDH*) in the GeneAmp PCR System 9700 (Applied Biosystems). Amplified DNA fragments were electrophoresed on 1.5% agarose gels containing ethidium bromide with a DNA molecular weight marker for comparison.

### Patients and specimens

This study included 233 patients with gastric adenocarcinoma invading deeper than the submucosal layer. All patients underwent curative gastrectomy with lymph node dissection at Kagoshima University Hospital from January 1995 to December 2004 (Table 1). In all, 75 patients underwent distal, 21 proximal, 124 total and 13 partial gastrectomy. A total of 233 gastric cancer patients were classified, including 161 male and 72 female patients (range, 31–85 years; average, 66 years). The final pathological examination disclosed that cases of stage I, II, III and IV gastric cancers numbered 70, 45, 63 and 55, respectively. The patients were histopathologically classified as 118 differentiated (papillary, well-differentiated and moderately differentiated tubular adenocarcinoma) or 115 undifferentiated (poorly differentiated adenocarcinoma, mucinous adenocarcinoma and signet-ring cell carcinoma) according to the Japanese classification of gastric cancer (Japanese Gastric Cancer Association, 1998). The study was approved by the Institutional Review Board of Kagoshima University and performed according to the Helsinki Declaration.

### Immunohistochemistry of BMP-7 in gastric cancer and its evaluation

The specimens of the gastric cancer were formalin-fixed and paraffin-embedded tissues; they were cut into 3-*μ*m-thick sections and mounted on glass slides for immunohistochemistry. They were deparaffinised in xylene and dehydrated with a series of graded ethanol. The endogenous peroxidase activity of specimens was blocked by immersing the slides in a 0.3% H_2_O_2_ solution in methanol for 30 min at room temperature. After washing three times with phosphate-buffered saline (PBS) for 5 min each, the sections were treated with 1% bovine serum albumin for 30 min to block nonspecific reactions at room temperature. The blocked sections were incubated with the mouse monoclonal antibody against human BMP-7 (1 : 500; R&D Systems, Inc., Minneapolis, MN, USA) and left 24 h at 4 °C, followed by staining with a streptavidin–biotin peroxidase kit (Vector Laboratories, Inc., Burlingame, CA, USA). The sections were washed in PBS for 5 min three times and the immune complex was visualised by incubating the sections with diaminobenzidine tetrahydrochloride. The sections were rinsed briefly in water, counterstained with haematoxylin and mounted. Noncancerous kidney samples were used as positive controls for BMP-7. Bone morphogenetic protein-7 expression was determined by counting the number of cancer cells in which the cytoplasm was stained with the anti-BMP-7 antibody. Evaluation of immunohistochemistry was independently carried out by two investigators (MA and SI). To evaluate this, 10 fields within the tumour were selected, and expression in 1000 cancer cells (100 cells per field) was evaluated using high-power ( × 200) microscopy. The average labelling index of BMP-7 was assessed according to the proportion of positive cells in each field. Bone morphogenetic protein-7 expression was graded as the BMP-7-positive group if >10% of cancer cells were stained or as the BMP-7-negative group if <10% of cancer cells were stained.

### Statistical analysis

A statistical analysis of group differences was performed using *χ*^2^ test. The Kaplan–Meier method was used for survival analysis and evaluated by the log-rank test. The Cox proportional hazard model was used in multivariate analysis. *P*<0.05 was considered statistically significant.

## Results

### BMP-7 expression in gastric cancer and its association with clinical results

**Figure 1:**
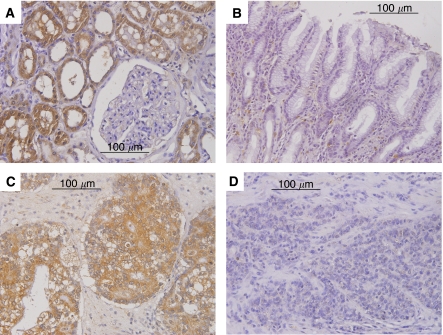
Expression of BMP-7 in clinical samples. Immunostaining of BMP-7 (original magnification, × 400): Examples of (**A**) noncancerous kidney tissue, (**B**) noncancerous gastric epithelium, (**C**) BMP-7-positive gastric cancer and (**D**) BMP-7-negative gastric cancer are shown. Staining is detected in cell membranes and the cytoplasm (**A**, **C**; they obviously stain brown).

**Figure 2:**
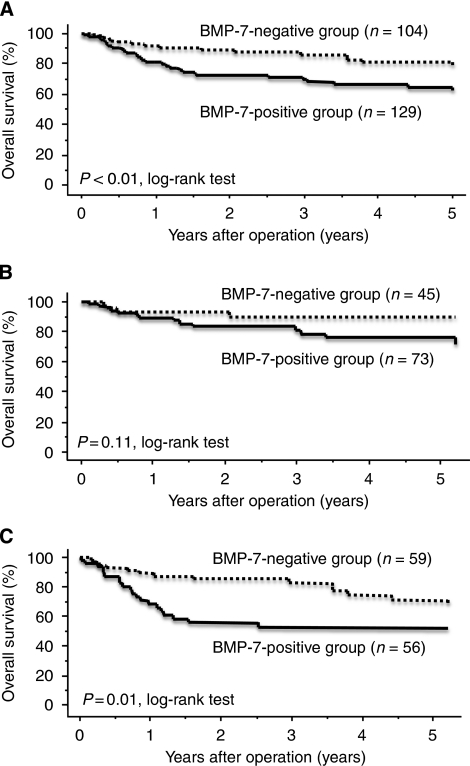
Postoperative survival curves of patients according to their expression of BMP-7 in gastric cancer. The BMP-7-positive group had significantly lower overall survival than the BMP-7-negative group (*P*<0.01, log-rank test) (**A**). In the differentiated group, no significant difference was observed in overall survival (*P*=0.11, log-rank test) (**B**). In the undifferentiated group, the BMP-7-positive group had significantly lower overall survival than the BMP-7-negative group (*P*=0.01, log-rank test) (**C**).

Immunohistochemically, in human gastric cancer, BMP-7 expression was identified in cellular membranes but also in the cytoplasm of cancer cells (Figure 1). According to the immunohistochemical evaluation, 129 of 233 patients (55%) were placed in the BMP-7-positive group. Bone morphogenetic protein-7 expression correlated with clinicopathological variables. Namely, tumour diameter, nodal involvement, lymphatic invasion and venous invasion were significantly greater in the BMP-7-positive group than in the BMP-7-negative group (*P*=0.01, <0.01, <0.01 and <0.01, respectively). Moreover, BMP-7 expression was significantly higher in the differentiated histology group than in the undifferentiated group (*P*<0.05). No significant difference was observed regarding age, gender or tumour depth (Table 2). The overall survival rate was significantly lower in the BMP-7-positive group than in the BMP-7-negative group (*P*<0.01; Figure 2A). Furthermore, we evaluated the correlation between expression of BMP-7 and prognosis in the differentiated and the undifferentiated histology groups. In the differentiated histology group, BMP-7 expression was not associated with postoperative outcome (Figure 2B). On the other hand, in the undifferentiated group, the BMP-7-positive group had significantly poorer survival than the BMP-7-negative group (*P*=0.01) (Figure 2C).

### Univariate and multivariate analyses of survival

Table 3 shows the results of univariate and multivariate analyses of factors related to patient prognosis. Univariate analysis showed that tumour histology, the depth of invasion, tumour size, lymph node metastasis, lymphatic invasion, venous invasion and BMP-7 expression were significantly related to postoperative survival (*P*<0.01). Multivariate analysis indicated that BMP-7 expression was one of the independent prognostic factors of overall survival for the patients with gastric cancer next to the depth of invasion and nodal involvement (*P*<0.01).

## Discussion

Bone morphogenetic protein-7 induces differentiation of mesenchymal cells to osteoblastic cells in bone and cartilage tissue (Ducy and Karsenty, 2000). Moreover, in kidney, it is related to MET and reverses chronic renal injury (Zeisberg *et al*, 2003). On the other hand, BMP-7 expression has been identified and clinical features of BMP-7 expression in several human cancers such as osteosarcoma, malignant melanoma, breast cancer, prostate cancer, colorectal cancer and renal cell cancer have been discussed (Sulzbacher *et al*, 2002; Masuda *et al*, 2004; Alarmo *et al*, 2006; Kwak *et al*, 2007; Rothhammer *et al*, 2007; Motoyama *et al*, 2008 ). In this study, robust expression of the *BMP-7* mRNA was identified by RT–PCR in MKN45 and MKN74 gastric cancer cell lines, which were derived from liver metastasis (data not shown).

We showed that BMP-7 expression was significantly associated with clinical factors such as lymphatic invasion, venous invasion and nodal involvement. Their previous studies demonstrated that BMP-7 promoted breast cancer cell migration and invasion, prostate cancer cell mobility and related metastasis in colorectal cancer (Yang *et al*, 2005; Grijelmo *et al*, 2007; Alarmo *et al*, 2009). In this context, BMP-7 expression may be connected with tumour aggressiveness beyond the specific organ. In this study, we showed that BMP-7 expression strongly correlated with nodal involvement. Thus, BMP-7 expression of biopsy may be informative to predict lymph node metastasis, when we should choose between endoscopic resection or gastrectomy for early gastric cancer.

As a prognostic parameter, Motoyama *et al* (2008) reported that overexpression of *BMP-7* mRNA was significantly associated with lower overall survival in colorectal cancer. Moreover, BMP-7 expression by immunohistochemistry was also significantly associated with lower recurrence-free survival in malignant melanoma and breast cancer (Rothhammer *et al*, 2007; Alarmo *et al*, 2008). We showed that BMP-7 expression in gastric cancer was an independent prognostic marker in accordance with results for other cancers. Only in renal cell carcinoma was BMP-7 expression significantly associated with better surgical outcome (Kwak *et al*, 2007). This may be explained by the fact that normal kidney cell usually highly expresses BMP-7 unlike normal gastric mucosa. Such differences might have caused the inverse result in prognostic implication.

In this study, BMP-7-positive gastric cancer was shown to be correlated with well-differentiated tumour histology. Andrews *et al* (1994) demonstrated that BMP-7-induced differentiation of pluripotent human embryonal carcinoma cells. Moreover, Lombardo *et al* (2011) reported that BMP-4-induced differentiation of colorectal cancer stem cells and increased their response to chemotherapy in mice. We demonstrated that BMP-7 expression was a more significant prognostic factor only in the undifferentiated group (Figures 2B and C). In short, although BMP-7 was highly expressed in the BMP-7-positive and undifferentiated group, the differentiation of this group was restricted, and the prognosis was poor. In the undifferentiated group, other factors that are associated with regulation of BMP signalling pathway might be associated with this result. The BMP signalling pathway is regulated in a complex manner by extracellular and intracellular factors and crosstalk with other signalling pathways, including TGF--signalling pathway (Zwijsen *et al*, 2003; Cao and Chen, 2005; Gazzerro and Canalis, 2006; Lombardo *et al*, 2011; Luo *et al*, 2010). It is necessary to further study the relationship between BMP signalling pathway and its related factors to open the view of chemotherapy in gastric cancer.

In conclusion, from the data collected, it would be appropriate to conclude on the possible regulation of gastric cancer progression by autocrine or paracrine BMP-7 loops. We can use BMP-7 expression as a predictor of lymph node metastasis and postoperative outcome in gastric cancer. As previously mentioned, the signals activated by BMP-7 are involve intracellular and extracellular factors, so further analysis seems to be necessary to determine the mechanism involved.

## Figures and Tables

**Table 1:** Patient characteristics

	**No.**
*Gender*	
Male	161
Female	72
	
*Age*	
Mean	66
Range	31–85
	
*Operation*	
Total gastrectomy	124
Distal	75
Proximal	21
Partial	13
	
*Stage*	
I	70
II	45
III	63
IV	55
	
*Histology*	
Differentiated	118
Undifferentiated	115

**Table 2:** Correlation between expression of BMP-7 and clinical factors

	**Expression of BMP-7**		
**Clinical factors**	**Positive (%) *n*=129 (55)**	**Negative *n*=104**	***P-*value**
*Age*			
<65	40 (48)	44	0.08
⩾65	89 (60)	60	
			
*Gender*			
Male	94 (58)	67	0.2
Female	35 (49)	37	
			
*Tumour size*			
<50 mm	40 (44)	50	0.01
⩾50 mm	89 (62)	54	
			
*Depth*			
T1 (sm)/T2 (mp)	85 (57)	65	0.68
T3 (ss)/T4 (se)	44 (53)	39	
			
*Nodal involvement*			
Yes	87 (63)	52	<0.01
No	42 (45)	52	
			
*Lymphatic invasion*			
Yes	107 (61)	69	<0.01
No	22 (39)	35	
			
*Venous invasion*			
Yes	88 (67)	44	<0.01
No	41 (41)	60	
			
*Histology*			
Differentiated	73 (62)	45	<0.05
Undifferentiated	56 (49)	59	

Abbreviation: BMP-7=bone morphogenetic protein-7.

**Table 3:** Univariate and multivariate analysis of prognostic factor in gastric cancer

**Clinical factors**	**Univariate *P***	**Multivariate *P***	**Hazard ratio**	**95% Confidence interval**
Age	0.4	—	—	—
Gender	0.88	—	—	—
Histology	<0.01	<0.05	1.893	1.021–3.510
Depth	<0.01	<0.01	3.239	1.650–6.361
Tumour size	<0.01	0.34	1.518	0.641–3.595
Nodal involvement	<0.01	<0.01	3.868	1.450–10.321
Lymphatic invasion	<0.01	—	—	—
Venous invasion	<0.01	0.18	1.171	0.555–2.474
BMP-7	<0.01	<0.01	2.934	1.524–5.648

Abbreviation: BMP-7=bone morphogenetic protein-7.
